# Neutral Mechanical Alignment and Prevalence of Constitutional Varus Knees Among the Saudi Population at King Saud University Medical City

**DOI:** 10.7759/cureus.41753

**Published:** 2023-07-12

**Authors:** Abdulaziz Almaawi, Fahad S AlAbdullatif, Abdullah H AlOmar, Bandar Aljammaz, Mohammed Almohaimeed, Abdulaziz Alkheraiji

**Affiliations:** 1 Department of Orthopedic Surgery, King Saud University, Riyadh, SAU; 2 Department of Orthopedics, King Khalid University Hospital, Riyadh, SAU; 3 College of Medicine, King Saud University Medical City, Riyadh, SAU; 4 Department of Orthopedics, Majmaah University, Riyadh, SAU

**Keywords:** osteoarthritis, deformity, radiology, constitutional varus, knee alignment

## Abstract

Background

Various studies have described the restoration of the neutral mechanical alignment of the lower limb in total knee replacement (TKR) as a key factor of knee implant durability. Mechanical alignment of the knee requires that the tibial and femoral cuts are perpendicular to the mechanical axis of each bone in the coronal plane.

Objectives

The aim of this study is to investigate mechanical knee alignment and the prevalence of constitutional varus knee among the Saudi population at a single tertiary center with no history of musculoskeletal abnormalities.

Methodology

This is a retrospective cross-sectional study involving patients with no musculoskeletal abnormality who had their lower limb X-ray Centigram using the universal viewer zero footprint system made between 2015 and 2021. The study took place at King Saud University Medical City. The X-rays were obtained from patients’ electronic medical records retrospectively. A total of 728 knees of 370 males and 358 females were included in this study.

Results

The results showed that 165 male knees (44.4%) and 218 female knees (60%) had constitutional varus alignment with the hip-knee-ankle (HKA) angle of ≤3° or less. The average HKA angle was smaller in males than in females: -3.69° versus -1.98°, respectively.

Conclusion

We have identified variables and factors that can help surgeons detect the constitutional varus on a full leg radiograph preoperatively at the time of TKA, regardless of the osteoarthritic changes of the knee. We encourage the scientific community to look for causes and risk factors for developing constitutional varus.

## Introduction

Mechanical alignment of the knee requires that the tibial and femoral cuts be perpendicular to the mechanical axis of each bone in the coronal plane. Putting this into consideration, we can say that the neutral mechanical alignment is characterized by a hip-knee-ankle (HKA) acute angle of 0° ± 3 [1].

Various studies have described the restoration of neutral mechanical alignment of the lower limb in total knee replacement (TKR) as a key factor in the durability of knee implants. The mechanical axis passes through the center of the knee joint in a neutrally aligned limb, reducing the risk of implant wear and component loosening [2,3]. Using intramedullary and extramedullary alignment rods or applying computerized navigation have been used to obtain intraoperative restoration of mechanical alignment. However, computerized navigation carries a major advantage in reducing mechanical axis and component positioning outliers [4].

There are many patients may exist for whom the neutral mechanical alignment is abnormal, like those who have constitutional varus knee (≥3° varus alignment). In order to acquire neutral mechanical alignment in such cases, a great extent of soft tissue release is unavoidable [3,4]. Moreover, limbs with less varus deformity preoperatively can develop a higher tendency to overcorrect into valgus postoperatively. That's why surgeons should avoid over-release of soft tissue medially in these cases to avoid such complications [5]. On the other hand, surgeons confront a dilemma when operating on a constitutional varus knee whether to restore it to neutral alignment or leave it in a slight varus alignment after surgery. Recently, a study has shown that leaving a varus knee at a slight varus after TKR leads to better functional outcomes than correcting it to neutral alignment [6].

Constitutional varus knees have been reported in many patients, especially the West with a prevalence of 32% in males and 17.2% in females [3]. Another study was performed among asymptomatic young individuals and found that 24% of the population have constitutional varus knee [7]. Patients with varus deformity of the knees >20° and femoral bowing >5° carry a greater risk of postoperative malalignment [5].

To our knowledge, there are no data documenting the rate of constitutional varus in the Saudi population. So, in this study, we aim to investigate mechanical knee alignment and determine the prevalence of constitutional varus knee among the Saudi population with no history of musculoskeletal abnormalities. Also, it is unclear how many of these patients can be recognized before developing knee osteoarthritis.

## Materials and methods

**Figure 1:**
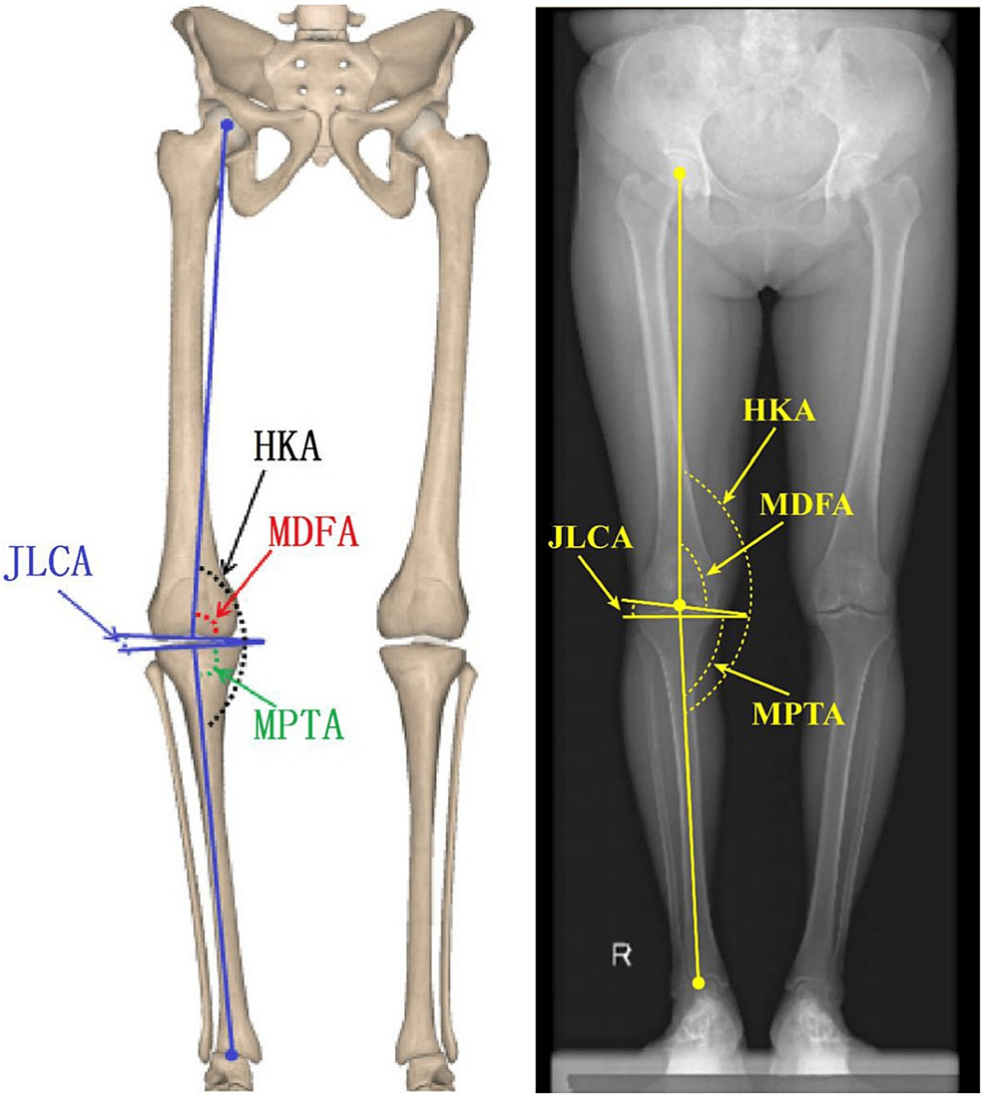
Schematic diagram of the mechanical angle of the lower extremity HKA = hip-knee-ankle (HKA); MDFA = distal medial femoral angle; MPTA = proximal medial tibial angle; JLCA = angle of joint line Source: [8]

This is a retrospective cross-sectional prevalence study including patients with lower limb x-ray Centigram between 2015 to 2021. The study took place at King Saud University Medical City. The research was approved by the institutional review board, with reference number 22/0168/IRB and named Natural Mechanical Alignment and Prevalence of Constitutional Varus Knee Among the Saudi Population at KSUMC. Radiographs were obtained from patients' electronic medical records. Figure 1 is a schematic diagram of the mechanical angle of the lower extremity.

From the literature, one study was found and used for power analysis [3]. Means and standard deviations from the study were used to estimate the required sample size using the mechanical lateral distal femoral angle using this formula. Using a reference population of 1,000 for males and females, 95% CI; the corrected calculated sample size was 201 males and 203 females. Thus, the total sample size of both sexes was 404. However, after excluding patients that haven’t met our criteria, we were left with 185 males, and 179 females, so our total sample was 364 patients. Data were gathered by measuring each knee separately, a total of 728 knees, 370 male knees and 358 female knees, were included.

All patients aged from 18 to 55 years old with a lower limb X-ray Centigram between 2015 to 2021, not known to have any musculoskeletal abnormality, were included in our study. For measurements, the angle formed by the mechanical femoral axis and the mechanical tibial axis was described as the hip-knee-ankle angle (HKA) angle. The lateral distal femoral angle (LDFA) was defined as the lateral angle between the mechanical axis of the femur and the distal femur joint line. The medial proximal tibial angle (MPTA) was defined as the medial angle between the mechanical axis of the tibia and the proximal tibia joint line. Moreover, the angle that is formed by the anatomic axis of the femur and the bisector of the femoral neck was described as the neck-shaft angle (NSA). The joint line convergence angle (JLCA) was the angle between the knee joint lines of the distal femur and proximal tibia. Nevertheless, the lateral proximal femoral angle (LPFA) was referred to as the angle between the straight line that connects the tip of the greater trochanter with the center of the femoral head and the mechanical femoral axis. Finally, the angle that was formed by the anatomical axis of the femur and the mechanical axis of the femur was called the valgus cut angle (VCA) [9,10].

A knee with an HKA angle between -3 and 3 was called a neutrally aligned knee, a constitutional varus knee if the HKA angle was -3 or less, and a constitutional valgus knee if the HKA angle was 3 or above.

Statistical analysis

An R2 was calculated based on the linear regression between the predicted values and the observed data. P values smaller than 5% were considered significant. Analysis was performed using the Statistical Package for Social Sciences (SPSS) version 23.0 (IBM Corp., Armonk, New York).

## Results

**Figure 2:**
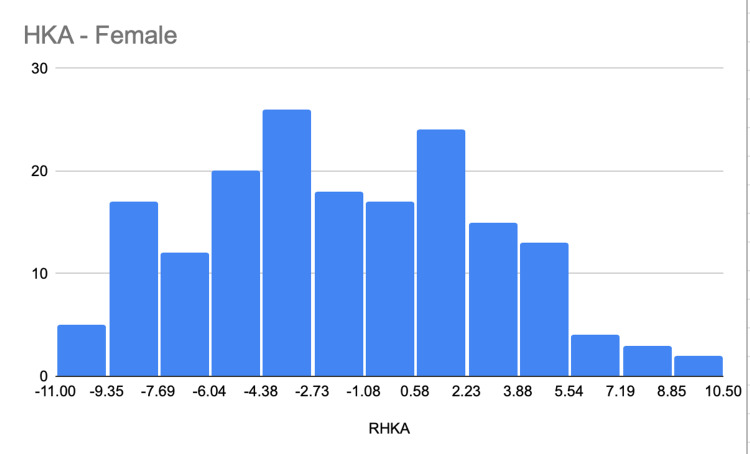
Hip-knee-ankle angle for females

**Figure 3:**
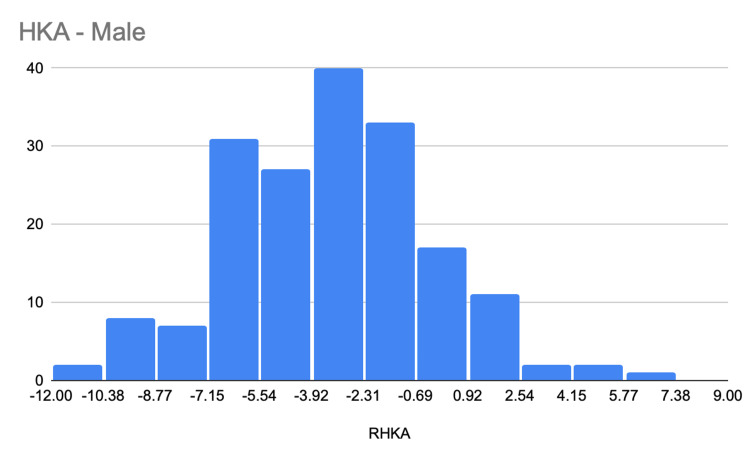
Hip-knee-ankle angle for males

The average HKA angle was significantly smaller in male than in female knees with an angle of -3.69° (SD, 3.34°) versus -1.98° (SD, 4.61°), respectively (P = 0.0001) (Figure 2, Figure 3). On the other hand. MPTA had a greater angle in females than in males, with an average difference of 2.01 ± 0.03. One-hundred sixty (160; 43.2%) of male knees and 127 (35.4%) of female knees had an HKA angle of between -3° and +3°. Thirty-four (9%) of the male and 13 (3%) of the female knees had an HKA angle of +3° or more (Table 1). 

**Table 1:** Measurement parameters for all knees and both genders separately The values are expressed as mean ± SD. HKA angle = hip–knee–ankle angle; LDFA = lateral distal femoral angle; MPTA = medial proximal tibial angle; JLCA = joint line convergence angle; NSA = neck–shaft angle; VPA = valgus proximal angle; LPFA = lateral proximal femoral angle.

Parameter	ALL (N = 728)	Male (N = 370)	Female (N = 358)	p value	
HKA	2.8555 ±4.10548	-3.6976 ±3.3460	-1.9852 ±4.61053	0.00001	
LDFA	87.7157 ±5.37298	87.7905 ±6.95822	87.6383 ±2.95517	0.208	
MPTA	87.3993 ±3.61593	86.4068 ±3.49055	88.4251 ±3.4577	0.832	
JLCA	1.7566 ±1.349)	1.4254 ±0.98556	2.0989 ±1.57185	0.00001	
NSA	126.55 ±7.8)	128.27000 ±7.536	124.77402 ±7.742	0.355	
VPA	5.54 ±0.97	5.4549 ±0.83766	5.6363 ±1.084	0.00001	
LFPA	89.24 ±7.10	88.886 ±5.76	89.61 ±8.261	0.526	

One hundred sixty-five (44.4%) of the male knees and 218 (60%) of the female knees had constitutional varus alignment with an HKA angle of -3° or less (Table 2). Furthermore, constitutional varus knees in females bilaterally represent 62 (17%) with 30 (8%) unilateral constitutional varus knees in females while men with bilateral constitutional varus represent 92 (25%) of the study population with 44 (12%) unilateral constitutional varus knee. Meanwhile, there were 276 (38%) knees with neutral alignment (-3° \ HKA \ 3°). In this group, the mean HKA was -0.9° (SD 1.53°) (Table 2). Moreover, eight (0.01%) of the asymptomatic male knees showed valgus alignment (HKA > 3°) of the lower limb with a mean HKA of 6.77° (SD 2.53°). while in females, asymptomatic valgus knee present 0.06% of the population with an average HKA of 5.45 (SD1.91) (Table 2). When controlled for sex, the greatest (R2 = .078 P = 0.001) contributor to constitutional varus was the MPTA. Other contributors were NSA (R2 = .050), HKA (R2 = .044), and JLCA (R2 = 0.011) (Table2).

**Table 2:** Parameters for constitutional varus knees in comparison to neutral and valgus knees The values are expressed as mean ±SD; * is the significant difference between varus and valgus knees (p = 0.05); § is the significant difference between varus and valgus knees (p = 0.001); ± is the significant difference between varus and neutral knees (p = 0.001); + is the significant difference between varus and neutral knees (p = 0.05); ~ is the significant difference between valgus and neutral knees (p = 0.05); & is the significant difference between valgus and neutral knees (p = 0.001). The values are expressed as mean ± SD. HKA angle = hip–knee–ankle angle; LDFA = lateral distal femoral angle; MPTA = medial proximal tibial angle; JLCA = joint line convergence angle; VPA = valgus proximal angle; NSA = neck–shaft angle; LPFA = lateral proximal femoral angle

PARAMETER	ALL (N=728)	Male			Female		
		Varus N=231	Neutral N=129	Valgus N=8	Varus N=160	Neutral N=147	Valgus N=48
HKA	2.8555 (SD = ±4.10548)	-5.65^§^ ± 1.96	-0.9^±^ ±1.53	6.77^&^ ± 2.53	6.16^§^ ±2.26	0.103^±^ ± 1.68	5.45^&^ ± 1.91
LDFA	87.7157 (SD = ±5.37298)	88.72^§^ ± 6.25	86.45^±^ ± 7.95	82.84^&^ ± 3.61	88.78^§^ ± 2.58	87.48^~^ ± 2.32	84.44^&^ ± 3.36
MPTA	87.3993 (SD = ±3.61593)	88.72^§^ ± 3.68	87.36 ± 2.73	89.58^&^ ± 3.57	87.64^§^ ± 4.08	88.80 ± 2.254	89.82^&^ ± 3.65
JLCA	1.7566 (SD = ±1.349)	1.45^§^ ± 1.01	1.36 ± 0.96	1.23 ± 0.92	2.74 ±1.80	1.53^±^ ± 1.11	1.69 ±1.06
NSA	126.55 (SD = ± 7.8)	128.49 ± 7.67	128.12^+^ ± 7.26	124.6^~^ ± 7.69	124.18 ± 7.35	125.70 ± 7.32	123.90 ± 9.84
VPA	5.54 (SD = ± 0.97)	5.51^*^ ± 0.842	5.36^±^ ± 0.81	5.24 ± 0.91	5.87 ± 1.02	5.38 ± 1.00	5.63 ± 9.84
LFPA	89.24 (SD = ±7.10)	88.79 ± 6.06	89.00 ±5.29	89.6 ± 4.49	89.85 ± 1.02	90.38 ± 5.35	86.53 ± 17.81

## Discussion

The neutral mechanical alignment of the lower limb is the base on which the success and durability of knee arthroplasty lean. It is the natural mechanical alignment that is considered by most surgeons as the normal situation of the knee, which is symmetric mediolateral joint loading. Mechanical alignment of the knee requires that the tibial and femoral cuts be perpendicular to the mechanical axis of each bone in the coronal plane. The neutral mechanical alignment is described as a hip-knee-ankle acute angle of 0° ± 3 [1,3].

Constitutional varus is thought to happen due to several reasons. Because of the limited evidence and data on this topic, a previous article has identified that gender has a great influence on having constitutional varus, where females are commonly affected more than males [10]. Furthermore, our study has shown that females are affected more than males. Apart from gender, other factors seem to influence the geometry of the knee as well, such as morphotypes, which were categorized as endomorph, mesomorph, or ectomorph depending on the individual's body [10,11].

The impact of constitutional varus on the patients is less likely to be documented as a majority of the patients are asymptomatic. According to Jan M. [12], the patient is usually asymptomatic when the HKA angle falls -1.3 with a range between -8.1° and 5.4° [7]. Constitutional varus is usually detected during postop with a follow-up lower limb X-ray, where you can see there is a degree of varus in the patient. Also, prolonged load on the medial aspect of the patient's knee causes erosion of the cartilage, which causes the patient to develop osteoarthritic changes and symptoms develop with time, making the patient seek medical help.

Yet other articles have shown that increased sports activity can be a risk factor for developing constitutional varus and the association of varus alignment with intense physical activity during growth has been marked as a factor by other studies [3].

Furthermore, several of the alterations that may affect knee varus development are influenced by the lifestyle of the Saudi population, such as sitting on the floor in a kneeling position, which may cause proximal tibial torsion and varus deformity [9]. Yet other studies have shown that increased sports activity can be a risk factor for developing a constitutional varus knee. Moreover, the association of varus alignment with intense physical activity during growth has been marked as a factor by other studies [3].

As mentioned before, a great number of surgeons try to restore the mechanical alignment as normal as possible, trying to reduce the risk of implant wear and component loosening [2,3]. On the other hand, to acquire natural mechanical alignment on a constitutional varus knee; a great extent of soft tissue release is needed [3,13]. That is why surgeons usually face a dilemma when operating on a constitutional varus knee. A study done recently concluded that returning such knees to slight varus is better than correcting it to natural mechanical alignment in terms of better functional outcomes [6]. According to Faschingbauer et al., in terms of varus/valgus deflection under load and tibial rotation, neutral alignment (0° tibia cut) was most similar to the constitutional varus-aligned knee [14].

Moreover, another study has shown that both postoperative mild varus and neutral mechanical alignment can give rise to good functional outcomes. It also suggested that slight residual postoperative varus is acceptable post-TKR for varus osteoarthritis [6]. According to Magnussen et al. [12], the remaining varus deformity following TKR had no negative effects on the 10-year survival rate or the International Knee Society score at a mean of 4.7 years after TKA in 553 patients [13]. Furthermore, Vanlommel et al. [3], found that the "mild varus" group had significantly better outcomes in the Western Ontario and McMaster Universities Osteoarthritis Index (WOMAC) score and the Knee Society Score (KSS) at the 7.3-year follow-up when comparing three groups (neutral, mild varus (0-3°), and varus (> 3°)) [14].

It was also noticed that the contributing variable that has the most influence on developing constitutional varus is MPTA. Which is similar to what Bellemans J et al. have published [3]. The other variables, such as NSA, joint line convergence angle, and LPFA relate to the increased varus angle, which confirms previously published work by Victor et al. [12]. reviewing the study by Parratte S et al. [7] on modern TKA concluded that there is no difference in the survival rate at 15 years of follow-up for knees aligned within the normal range of neutral angle ± 3° versus outliers [15]. These finding made other authors think that every individual-specific dynamic in gait and loading play a significant role.

Our study had some limitations. First, it is a retrospective cross-sectional study. Observations were taken by three co-authors for all the measurements, where accuracy can differ from one to another. The second limitation of our study was the use of an X-ray Centigram for the measurements, although this method is well-validated, the accuracy of the radiographic technicians who were assigned to perform the X-ray Centigram can change from one patient to another; another option might have been to use CT scans, which could help in visualizing full anatomical landmarks.

## Conclusions

In conclusion, our findings demonstrate that a considerable proportion of the normal Saudi population (44.4% of males and 60% of women) has varus alignment predominantly in females. We have also identified some variables and factors, which can help and serve surgeons to detect constitutional varus on a full-leg radiograph at the time of TKA, regardless of the osteoarthritic changes of the knee. Finally, we encourage the scientific community to look for causes and risk factors for developing constitutional varus.
